# From the Outside-In: The *Francisella tularensis* Envelope and Virulence

**DOI:** 10.3389/fcimb.2015.00094

**Published:** 2015-12-23

**Authors:** Hannah M. Rowe, Jason F. Huntley

**Affiliations:** Department of Medical Microbiology and Immunology, University of Toledo College of Medicine and Life SciencesToledo, OH, USA

**Keywords:** *Francisella*, tularemia, tularensis, outer membrane proteins, virulence factors, envelope

## Abstract

*Francisella tularensis* is a highly-infectious bacterium that causes the rapid, and often lethal disease, tularemia. Many studies have been performed to identify and characterize the virulence factors that *F. tularensis* uses to infect a wide variety of hosts and host cell types, evade immune defenses, and induce severe disease and death. This review focuses on the virulence factors that are present in the *F. tularensis* envelope, including capsule, LPS, outer membrane, periplasm, inner membrane, secretion systems, and various molecules in each of aforementioned sub-compartments. Whereas, no single bacterial molecule or molecular complex single-handedly controls *F. tularensis* virulence, we review here how diverse bacterial systems work in conjunction to subvert the immune system, attach to and invade host cells, alter phagosome/lysosome maturation pathways, replicate in host cells without being detected, inhibit apoptosis, and induce host cell death for bacterial release and infection of adjacent cells. Given that the *F. tularensis* envelope is the outermost layer of the bacterium, we highlight herein how many of these molecules directly interact with the host to promote infection and disease. These and future envelope studies are important to advance our collective understanding of *F. tularensis* virulence mechanisms and offer targets for future vaccine development efforts.

## Introduction

*Francisella tularensis* is a Gram-negative intracellular bacterium and the causative agent of the zoonotic disease tularemia (Carvalho et al., 2014). *F. tularensis* has been further subdivided into two subspecies: *F. tularensis* subsp. *tularensis*, also referred to as Type A strains which are found exclusively in North America; and *F. tularensis* subsp. *holarctica*, also referred to as Type B strains which are found throughout the northern hemisphere (Oyston et al., 2004; Keim et al., 2007). Type A and Type B strains are extremely infectious to humans and can be acquired through various routes, including arthropod bites, contact with infected animals, ingestion of contaminated food or water, or inhalation of aerosols (Kingry and Petersen, 2014). Depending on the route of infection, various organs and tissues are heavily colonized by *F. tularensis*, including skin, lymph nodes, lungs, spleen, liver, and kidney, with bacteremia common in the early stages of infection. Disease onset is typically very rapid with flu-like symptoms common, including fever, headache, chills, malaise, and sore throat (Dennis et al., 2001).

Because of its low infectious dose, multiple routes of infection, and high morbidity and mortality rates, *F. tularensis* has been designated as a Tier 1 Select Agent by the U.S. Centers for Disease Control and Prevention (CDC), highlighting concerns over its potential use a bioterrorism agent (Dennis et al., 2001). Type A strains are the most virulent (ID_50_ < 10 CFU via multiple infection routes in many animals, including humans; Ellis et al., 2002; Molins et al., 2010), with the human ulcer isolate, Schu S4, being the most commonly-used strain in BSL3 laboratories (Molins et al., 2014). Type B strains are highly-infectious to mice and guinea pigs (pulmonary and intradermal ID_50_ < 10 CFU; Ellis et al., 2002; Molins et al., 2010) but higher doses are needed to infect rabbits (10^6^–10^9^ CFU subcutaneously) and humans (< 10^3^ via multiple routes; Ellis et al., 2002; Petersen and Molins, 2010). Despite these differences in virulence, Type B strains cause substantially more infections worldwide (Petersen and Molins, 2010; Hestvik et al., 2015). An attenuated Type B strain, the live vaccine strain (LVS), was developed in the former Soviet Union by serial passage through mice (Eigelsbach and Downs, 1961). Despite its name, LVS is not licensed for human vaccination in the U.S. due to safety and efficacy concerns (Oyston, 2009). However, LVS has been extensively used in research laboratories because of its ability to be safely used in BSL2 environments, high virulence in mouse models, high degree of genetic conservation with virulent Type A and Type B strains, and similar intracellular replication kinetics as virulent Type A and Type B strains (Elkins et al., 2007; Jones et al., 2014). A genetically-related species, *Francisella novicida*, also can be manipulated at BSL2 and does not cause disease in healthy humans, but *F. novicida* has a number of genetic and phenotypic differences that bring into question its use as a *F. tularensis* surrogate (Kingry and Petersen, 2014). The purpose of this review is to summarize the current knowledge on *F. tularensis* virulence factors. As such, we made every attempt throughout this review to clearly note what strain was used in each of the referenced studies so that readers can render their own judgments about the applicability to human disease.

Whereas, *F. tularensis* is a significant pathogen based on high morbidity and mortality rates, it also is an extremely interesting pathogen due its complicated intracellular lifestyle, ability to infect a wide variety of host cell types, persistence in the environment, and lack of classical bacterial virulence factors such as exotoxins or a Type III secretion system (Celli and Zahrt, 2013). Many excellent reviews previously have characterized *F. tularensis* as a “stealth” pathogen, which first evades host immune detection (Sjöstedt, 2006; Jones et al., 2014), but subsequently induces a cytokine storm that causes host death (Cowley, 2009; Cowley and Elkins, 2011). In addition, the metabolic pathways and nutrient requirements of *F. tularensis* promoting survival inside host cells also have been elegantly outlined (Barel and Charbit, 2013; Barel et al., 2015). Here, we will highlight studies that have identified and characterized more “classical” virulence factors of *F. tularensis* (i.e., those bacterial molecules that directly interact with the host, directly damage the host, or sense changes in the environment to modify bacterial gene expression). This review will begin with the outermost capsular layer and sequentially discuss the roles of LPS, the outer membrane, periplasm, and inner membrane in *F. tularensis* virulence and disease.

## Capsule

Polysaccharide capsules are produced by bacteria such as *Escherichia coli, Neisseria meningitidis*, and *Streptococcus pneumoniae* (Preston and Dockrell, 2008; Willis and Whitfield, 2013), whereas protein capsules are produced by bacteria such as *Bacillus anthracis* (Cote et al., 2011). Capsule typically protects bacteria from complement-mediated lysis, phagocytosis, and immune recognition. For intracellular pathogens like *F. tularensis*, antiphagocytic properties ostensibly may appear counterproductive, but it is now well-appreciated that “stealth” pathogens manipulate host cell entry and intracellular trafficking mechanisms to limit inflammation and promote intracellular survival (Geier and Celli, 2011; Dai et al., 2013). Conversely, capsule can elicit protective immunity, as evidenced by successful capsule-based commercial vaccines against *S. pneumoniae, Haemophilus influenza*, and *N. meningitidis* (McIntyre et al., 2012). Given the disparate roles of bacterial capsule in virulence and protective immunity, *Francisella* capsule has been the focus of many research studies.

An electron-transparent, capsule-like outer structure, approximately 0.02–0.04 μm thick, was first described by Hood and shown to play a role in *F. tularensis* virulence (Hood, 1977). Capsule was observed in *F. tularensis* strain Schu S4 when bacteria were aerosolized or grown in modified casein hydrolysate liquid medium. However, capsule was absent from bacteria exposed to air long-term (20 h), treated with 10% sodium chloride, or taken from aged cultures (6 months at 4°C). Biochemical analyses indicated that *F. tularensis* capsule was distinct from cell wall material, being composed of ~51% lipids (primarily 14 and 16 carbon length fatty acids), up to 35% amino acids, and up to 21% carbohydrates (including mannose and rhamnose; Hood, 1977). Capsule production appears to be well-conserved among many *F. tularensis* strains, as a monoclonal antibody generated against Schu S4 capsular material cross-reacted with 14 different Type A and Type B isolates, including LVS, but did not react against LPS (Apicella et al., 2010). Detailed analysis of the *F. tularensis* capsule revealed polysaccharide identical to the O-antigen subunit of LPS that diffusely migrated on SDS-PAGE between 100 and 250 kDa (Apicella et al., 2010). As proof that *F. tularensis* capsule was composed of O-antigen and no other LPS components, mass spectrometry and NMR analyses demonstrated that purified capsule lacked other LPS-specific components, including 2-keto-3-deoxyoctulsonic acid (KDO) and lipid A (Apicella et al., 2010; Bandara et al., 2011). Given the large molecular weight and identical nature of *F. tularensis* capsule O-antigen and LPS O-antigen, *F. tularensis* capsule appears to be a Group 4 capsule (Whitfield, 2006). As noted above, whereas Schu S4 capsule was found to be composed of mannose and rhamnose, a LVS capsule-like complex was found to contain mannose, glucose, and galactose, indicating that capsular composition may differ between Type A and B species (Bandara et al., 2011). Together, these studies demonstrated that many *F. tularensis* strains express a polysaccharide capsule that it is identical to the O-antigen but lacks other LPS components.

Capsule has been shown to play an important role in *F. tularensis* virulence both *in vitro* and *in vivo*. For Schu S4, acapsular bacteria were attenuated when guinea pigs were infected intraperitoneally (i.p.) or when mice were infected either i.p. or intranasally (i.n.) (Hood, 1977; Lindemann et al., 2011; Rasmussen et al., 2014). In macrophages, Schu S4 acapsular mutants exhibited reduced cytosolic growth following phagosomal escape, due to the triggering of apoptotic or pyroptotic cell death (Lindemann et al., 2011). That study highlighted the importance of capsule to evade intracellular detection pathways. Capsule also contributes to LVS virulence, as serial passage on Chamberlain's medium increased LVS capsule production and capsulated LVS was substantially more virulent in mice via intravenous (i.v.), i.p., or i.n. routes (Cherwonogrodzky et al., 1994). Similarly, acapsular LVS mutants were found to be attenuated in macrophages and mice infected either i.p. or i.n. (Sandström et al., 1988; Bandara et al., 2011).

Whereas bacterial capsules typically block complement or antibody from binding to the bacterial surface, the role of *F. tularensis* capsule in serum resistance is less clear. In one study, chemically-mutagenized LVS rough colonies that lacked electron-dense surface layers (i.e., acapsular mutants) were susceptible to killing by non-immune human serum, primarily through the binding of IgM and complement component C3 (Sandström et al., 1988). A second study noted that *F. tularensis* spontaneous acapsular mutants absorbed more human serum components than wild-type capsulated strains and this effect primarily was mediated by IgM (Sorokin et al., 1996). Finally, Schu S4 acapsular mutants were found to be serum sensitive (Lindemann et al., 2011). While these three studies suggest that *F. tularensis* capsule blocks antibody and complement binding to the bacterial surface, other studies have suggested that serum resistance cannot be solely attributed to capsule. For example, an acapsular LVS mutant (Δ*FTL1422–1423*) was found to be completely resistant to both guinea pig and human serum (Bandara et al., 2011). In addition, a number of studies have noted antibody and/or complement binding to LPS (see Lipopolysaccharide Section). Based on these conflicting results, further studies are needed to better understand the role of capsule in serum resistance.

There has been much interest in identifying the genes involved in *F. tularensis* capsule production. However, given the above referenced studies noting that *F. tularensis* capsule and O-antigen polysaccharides are identical, it is not surprising that capsule gene identification studies have been difficult to interpret or have yielded conflicting results. A detailed analysis of gene deletion mutants indicated that *wbtA1, wbtA2, wbtC, wbtI, wbtM*, and *FTL0708* were required for capsule production, whereas *capB, capC, lpxL, wbtK, FTL0706*, and *FTT0673–FTT0674* were not (Apicella et al., 2010; see Table 1 for corresponding gene loci in Schu S4, LVS, or *F. novicida*). Of those genes, individual deletions of *wbtA1, wbtA2, wbtC, wbtI*, or *wbtM* resulted in loss of both capsule and O-antigen, highlighting that capsule and O-antigen share some biosynthetic pathway components. Conversely, only the *FTL0708* mutant was found to be deficient in capsule production but not LPS, demonstrating that some genes may be capsule-specific. In a separate study, a LVS glycoprotein-containing capsule-like complex was linked to loci *FTL1421–1432* and deletion of both *FTL1422* and *FTL1423* resulted in loss of this capsule-like complex (Bandara et al., 2011). In Schu S4, loci *FTT1236–1238* was shown to be responsible for both O-antigen production and capsule biosynthesis, of which *FTT1236 (waaY)* and *FTT1238 (waaL)* were specifically required for Schu S4 capsule production (Lindemann et al., 2011). Whereas, the aforementioned studies focused on polysaccharide or O-antigen-related genes, a three gene locus named *capBCA*, homologous to the *B. anthracis* poly-γ-D-glutamic acid capsule *cap* locus, was found to be required for LVS i.n. virulence in mice (Su et al., 2007). The *capBCA* locus is conserved across Type A and B strains, and mutagenesis studies in Schu S4 and LVS confirmed that *capBCA* or *capB* mutants were attenuated both *in vitro* and *in vivo* (avirulent by subcutaneous [sub-q], intradermal [i.d.], and i.n. routes; Su et al., 2007; Jia et al., 2010; Michell et al., 2010). However, capsule production was not assessed in any of those virulence studies, bringing into question if the *capBCA* locus truly is involved in *F. tularensis* capsule production. Indeed, as noted earlier, Apicella et al., found that *capB* and *capC* were not involved in LVS capsule production, suggesting that the *cap* genes perform another function. A more recent study suggested that *capA* may encode an integral inner membrane protein that interacts with CapB and CapC to export carbohydrates extracellularly (Martin-Garcia et al., 2014). Given the large number of genes and operons that have been reported to play roles in *F. tularensis* capsule production, more studies are clearly needed.

**Table 1:** ***Francisella* genes involved in capsule and/or LPS synthesis**.

**Gene name (if designated)**	**Schu S4 locus**	**LVS locus**	***F. novicida* strain U112 locus**	**Capsule, LPS, or both**
*lpxD2*	FTT0286c	FTL0196	FTN0200	LPS
*flmF2*	FTT0454	FTL1611	FTN0545	LPS
*flmK*	FTT0455c	FTL1609	FTN0546	LPS
	FTT0789	FTL1432	FTN1221	Capsule
	FTT0790	FTL1431	FTN1220	Capsule
	FTT0791	FTL1430	FTN1219	Capsule
	FTT0792	FTL1429	FTN1218	Capsule
	FTT0793	FTL1428	FTN1217	Capsule
	FTT0794	FTL1427	FTN1216	Capsule
	FTT0795	FTL1426	FTN1215	Capsule
	FTT0796	FTL1425	FTN1214	Capsule
	FTT0797	FTL1424	FTN1213	Capsule
	FTT0798	FTL1423	FTN1212	Capsule
	FTT0799	FTL1422	FTN1211	Capsule
	FTT0800	FTL1421	FTN1210	Capsule
*capA*	FTT0805	FTL1416	FTN1201	unknown
*capC*	FTT0806	FTL1415	FTN1200	unknown
*capB*	FTT0807	FTL1414	FTN1199	unknown
*lpxE*	FTT0891	FTL0393	FTN0416	LPS
*waaY*	FTT1236	FTL0708	FTN1254	Capsule
*waaZ*	FTT1237	FTL0707	FTN1255	LPS
*waaL*	FTT1238	FTL0706	FTN1256	Capsule
*wbtM*	FTT1450c	FTL0606	FTN1416	Both
*wzx*	FTT1453c	FTL0603	FTN1419	LPS
*wbtI*	FTT1455c	FTL0601	FTN1421	Both
*wzy*	FTT1458c	FTL0598	FTN1424	LPS
*wbtF*	FTT1459c	FTL0597	FTN1425	LPS
*wbtE*	FTT1460c	FTL0596	FTN1426	LPS
*wbtD*	FTT1461c	FTL0595	FTN1427	LPS
*wbtC*	FTT1462c	FTL0594	FTN1428	Both
*wbtA2*	FTT1463c	FTL0593	FTN1430	Both
*wbtA1*	FTT1464c	FTL0592	FTN1431	Both
*kdtA*	FTT1561	FTL0547	FTN1469	LPS
*lpxD1*	FTT1571c	FTL0537	FTN1480	LPS
*lpxF*	FTT1643c	FTL1815	FTN0286	LPS

The surface localization of bacterial capsules often has made them attractive targets for vaccine development efforts. However, *Francisella* capsular material has yielded mixed results as a protective antigen. Schu S4 capsular material did not elicit protection in guinea pigs or mice against i.p. Schu S4 challenge (Hood, 1977). However, immunization with LVS capsular material protected mice from i.p. LVS challenge, despite not protecting mice from i.p. Schu S4 challenge (Apicella et al., 2010). The use of capsule-deficient strains as live attenuated vaccines has yielded more promising results, with a LVS Δ*FTL1422–1423* strain protecting against high-dose wild-type i.n. LVS challenge (Bandara et al., 2011) and Schu S4 Δ*waaY* and Δ*waaL* strains protecting against wild-type Schu S4 i.p. and i.n. challenge (Rasmussen et al., 2014). Importantly, the use of capsule-deficient mutants as vaccine strains indicates that *F. tularensis* capsule may not be a protective antigen but that capsule-deficient mutants are highly-attenuated and could be safe and effective vaccine candidates (Bandara et al., 2011). Despite the unlikely role of the *capBCA* locus in capsule production, a LVS *capBCA* mutant protected mice from wild-type i.n. LVS challenge (Su et al., 2007). Similarly, both LVS and Schu S4 Δ*capB* mutants protected mice against Schu S4 aerosol and sub-q challenge (Jia et al., 2010; Michell et al., 2010).

**Figure 1:**
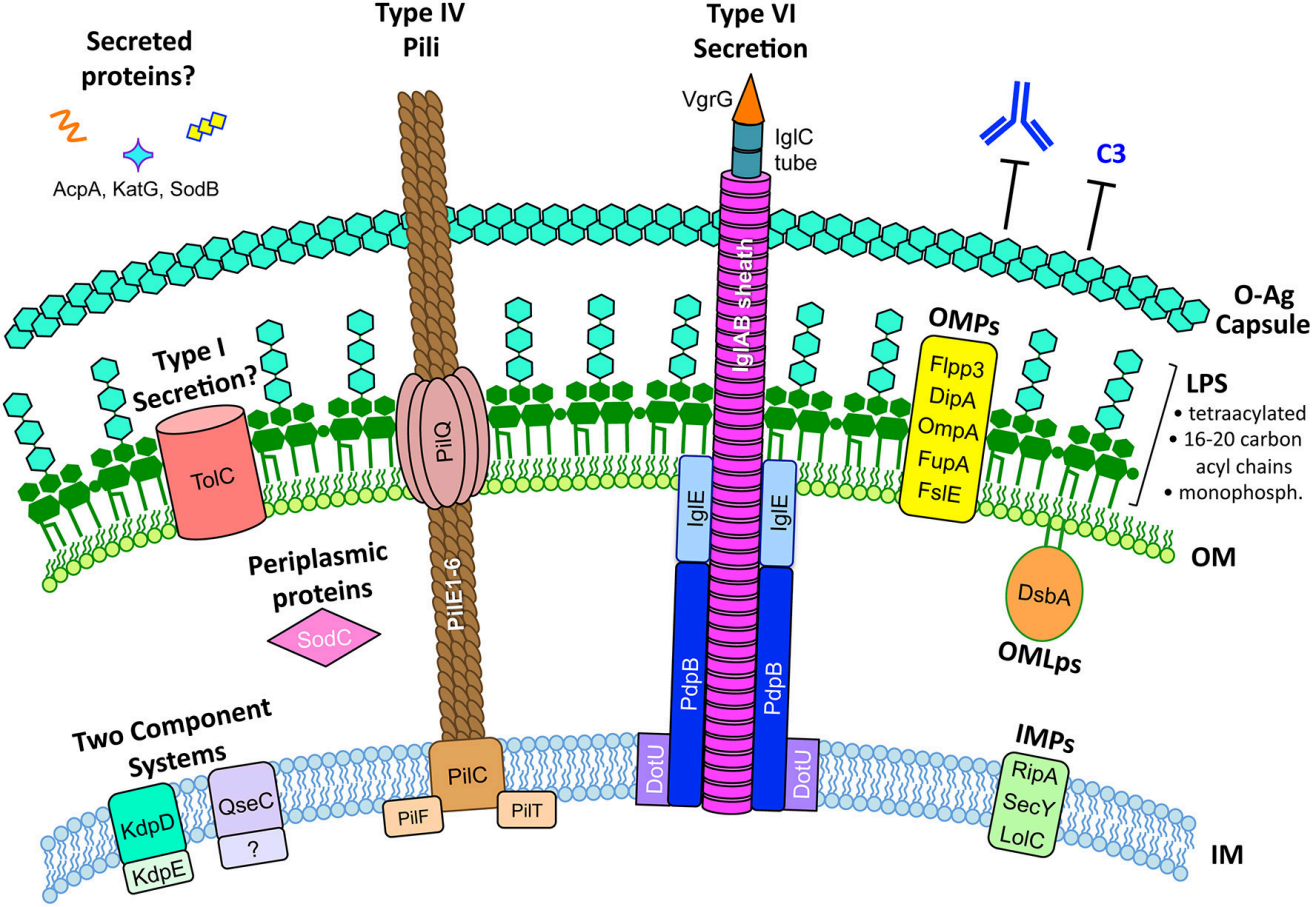
**The *F. tularensis* envelope and its role in virulence**. The *F. tularensis* capsule, identical to the O-antigen of LPS, blocks IgM and complement component C3 binding. *F. tularensis* LPS differs from prototypic LPS in that it is tetraacylated, possesses long-chain acyl chains (16–20 carbons long), is either non- or mono-phosphorylated, and is a weak TLR4 agonist. Type IV pili form horn-like protrusions on the bacterial surface and, while correlated with virulence, the exact function of *F. tularensis* type IV pili are not known. A *Francisella* Pathogenicity Island (FPI)-encoded Type VI-like secretion system has been reported in *F. novicida* (Table 3) but expression and function in virulent *F. tularensis* strains has not been reported. Various proteins have been reported to be secreted by *F. tularensis*, including AcpA, KatG, and SodB (Table 3), but the mechanisms for their secretion are unknown. The *F. tularensis* outer membrane (OM) contains many outer membrane proteins (OMPs) and outer membrane lipoproteins (OMLps) that are required for virulence (Table 4). Periplasmic and inner membrane proteins (IMPs) also play roles in *F. tularensis* virulence (Table 5), but much less is known about these envelope compartments. See individual sections for specific details and references.

In summary, a majority of studies have demonstrated that *F. tularensis* produces a polysaccharide capsule that is identical to the O-antigen and that capsule is required for *F. tularensis* virulence (Figure 1). Despite a number of studies demonstrating that capsule is required for serum resistance, some controversy remains about the true role of capsule in serum resistance, specific composition of the *F. tularensis* capsule, and the genes required for capsule production. In addition, some aberrant findings suggest that multiple capsule-like substances may be produced by different *Francisella* strains and that capsule composition might vary depending on culture conditions or environmental stimuli. If various capsule compositions do indeed exist, these capsule variations may be an important part of the overall virulence strategy of *Francisella*.

## Lipopolysaccharide

Lipopolysaccharide (LPS) makes up the outer leaflet of the outer membrane for most Gram-negative bacteria. LPS is composed of three main components; lipid A, core oligosaccharide, and the O-antigen (Miller et al., 2005). Of these, lipid A (endotoxin) is highly immunostimulatory and lipid A modifications are a common virulence strategy for many Gram-negative pathogens (Tan and Kagan, 2014). *E. coli* lipid A is the prototype for many Gram-negative lipid A structures, with six acyl chains (hexaacylated) that are 12–14 carbons in length. By comparison, the atypical lipid A of *Francisella* is tetraacylated with fatty acid chains 16–18 carbons in length (Vinogradov et al., 2002; Phillips et al., 2004). These *F. tularensis* LPS modifications, and others described below, greatly reduce TLR4 activation and allow for immune evasion.

A long-recognized *F. tularensis* blue-gray colony variation has been shown to correlate with differences in virulence, host cell stimulation, immunogenicity, and alterations in LPS (Eigelsbach et al., 1951; Hartley et al., 2006; Soni et al., 2010). In fact, the highly-virulent Type A strain Schu S4 owes its “S4” designation to the “S_4_” smooth blue colony phenotype, as compared to the “S_1_” and “S_2_” smooth buff-colored colony phenotypes, or “NS” non-smooth gray colony phenotypes that were isolated from this clinical isolate (Eigelsbach et al., 1951). In i.p. mouse infection studies, smooth blue Schu S_4_ variants were extremely virulent whereas non-smooth gray Schu variants were six-logs attenuated (Eigelsbach et al., 1951). Gray variants also have been reported in LVS and these spontaneously-occurring colony variants were attenuated in rat macrophages due to their induction of high levels of nitric oxide (NO), as compared with wild-type LVS which replicated to high numbers in rat macrophages and induced very little NO (Cowley et al., 1996). Differences in virulence and macrophage activation appeared to be due to alterations in lipid A, as purified lipid A from the LVS gray variant induced more NO than lipid A from wild-type LVS. In that same study, *F. novicida* whole bacteria or purified lipid A from *F. novicida* were found to induce more NO than either wild-type LVS bacteria or gray variant LVS bacteria, demonstrating that *F. novicida* LPS is distinct from *F. tularensis* LPS (Cowley et al., 1996). More recent work on gray variant LVS determined two key features: first, gray variants express less LPS O-antigen than wild-type LVS; second, gray variants have reduced lipid A galactosamine modifications compared to wild-type LVS. Glycosyltransferase genes *flmF2 (FTL_1611)* and *flmK (FTL_1609)* were found to be expressed at significantly lower levels in gray-variant LVS and deletion of *flmF2* from wild-type LVS reduced the frequency of phase variation, supporting a role for these glycosyltransferases in phase variation (Soni et al., 2010). Finally, whereas wild-type LVS protected mice against Schu S4 i.n. challenge, LVS gray variants could not, indicating that O-antigen expression levels and lipid A galactosamine modifications likely are important for immunogenicity.

Detailed NMR and mass spectrometry studies have revealed that LPS O-antigen polysaccharide repeats are identical between Schu S4 and LVS, but differ from *F. novicida* LPS (Prior et al., 2003; Vinogradov and Perry, 2004; Thomas et al., 2007; Wang et al., 2011). The differences in LPS O-antigen are believed to be due to genomic differences in the *wzy, wzx*, and *wbt* loci, with Schu S4 and LVS both containing 15 O-antigen genes, as compared to *F. novicida* which contains 12 O-antigen genes (Prior et al., 2003; Thomas et al., 2007). For all *Francisella* strains, the loci are flanked by transposases and have a lower G-C content than the rest of the *Francisella* genome, suggesting possible horizontal gene acquisition of the O-antigen operon. Deletion of three highly-conserved LPS O-antigen genes, *wbtDEF*, in *F. novicida* and *F. tularensis* Schu S4 resulted in attenuation in mice via a sub-q infection route and loss of LPS O-antigen production in both bacterial species. However, despite the mutual loss of LPS O-antigen, only *F. novicida wbtDEF* mutants were serum sensitive and only Schu S4 *wbtDEF* mutants were attenuated for growth in mouse macrophages (Thomas et al., 2007). In contrast, whereas LVS *wbtDEF* mutants also were found to be LPS O-antigen deficient and serum sensitive, LVS *wbtDEF* mutants were not attenuated for human macrophage replication (Clemens et al., 2012). Taken together, these results indicate that although the *Francisella* LPS O-antigen is generally required for virulence in animals, the LPS O-antigen may play subtly different roles in intracellular survival and serum resistance among the different *Francisella* species. Importantly, LPS O-antigen composition appears to be critical for protective immunity, as *F. novicida* LPS protects against i.p. *F. novicida* challenge, LVS LPS protects against sub-q LVS challenge, and Schu S4 LPS protects against sub-q LVS challenge. In contast, *F. novicida* LPS does not protect against sub-q LVS or sub-q Schu S4 challenge and Schu S4 LPS does not protect against i.p. *F. novicida* challenge (Fulop et al., 2001; Thomas et al., 2007). The importance of LPS O-antigen in stimulating protective immune responses was further highlighted by the fact that vaccination with a *F. novicida wbtDEF* mutant was unable to protect mice from wild-type *F. novicida* i.p. challenge and a SchuS4 *wbtDEF* mutant was unable to protect mice from wild-type Schu S4 sub-q challenge (Thomas et al., 2007). Follow-up studies have confirmed that Schu S4 *wbtC* and *wbtI* also play roles in LPS O-antigen synthesis, as both mutants had altered or missing LPS O-antigen, were attenuated in mice infected i.d., and did not protect against low dose Schu S4 aerosol challenge. In addition to its role in LPS O-antigen synthesis, *wbtC* also appears to play a role in protein glycosylation, as *wbtC* mutants displayed fewer glycosylated isoforms of both DsbA (see Outer Membrane Proteins Section) and PilA (see Type IV Pili Section), which previously were reported to be glycosylated in *F. tularensis* (Twine et al., 2012).

In most Gram-negative bacteria, lipid A is linked to the core region through two KDO molecules (Okan and Kasper, 2013). By comparison, the *F. tularensis* core region contains only one KDO unit (Vinogradov et al., 2002; Vinogradov and Perry, 2004). Deletion of the putative core oligosaccharide KDO transferase gene, *kdtA*, from Schu S4 resulted in loss of the core oligosaccharide. Although, *kdtA* mutants still produced O-antigen, it was not present on the bacterial surface, supporting the role of *kdtA* in core oligosaccharide synthesis. In agreement with other studies demonstrating the importance of LPS in virulence and immunogenicity, Schu S4 *kdtA* mutants were avirulent in mice via an i.d. infection route but were not protective against Schu S4 aerosol challenge (Twine et al., 2012).

The lipid A component of *Francisella* LPS is conserved across species but possesses key differences from conventional LPS. First, *Francisella* lipid A is tetraacylated, as compared to the hexaacylated lipid A commonly found in most Gram-negatives. Second, *Francisella* lipid A acyl chains are 16 to 18 carbons long, as compared to the 12–14 carbon length chains of conventional LPS. Third, *Francisella* lipid A is either non-phosphorylated or mono-phosphorylated (at the 1′ position), as compared to prototypic lipid A which is phosphorylated at the 1′ and 4′ positions of the diglucosamine backbone. Fourth, *Francisella* masks its 1′ phosphate, if present, by adding galactosamine. The reduction or absence of negatively-charged phosphates likely affects the overall charge of *Francisella* LPS and confers resistance to antimicrobial peptides (Gunn and Ernst, 2007; Okan and Kasper, 2013). More comprehensive analyses of *Francisella* lipid A have revealed that a variety of structures exist, including acyl chains of varying lengths (from 14 to 20 carbons) and various sugar substitutions at the 1′ and 4′ backbone positions (Schilling et al., 2007; Beasley et al., 2012). Orthologs of the nine *E. coli* lipid A synthesis enzymes exist in *F. tularensis*, indicating that nascent *Francisella* lipid A is synthesized with phosphate groups at both 1′ and 4′ positions. However, *Francisella* actively dephosphorylates its lipid A at the 1′ position using the LpxE phosphatase and at the 4′ position using the LpxF phosphatase (Wang et al., 2004, 2006). The importance of lipid A desphosphorylation as a virulence mechanism is highlighted by studies demonstrating that *F. novicida lpxF* mutants are avirulent in macrophages and mice (aerosol, i.d., and sub-q infection routes tested) and are hypersensitive to antimicrobial peptides (Wang et al., 2007; Kanistanon et al., 2012). As noted above, LPS appears to generate species-specific immune responses, as the *F. novicida* Δ*lpxF* strain protected mice against wild-type i.p. or aerosol *F. novicida* challenge but did not confer protection against virulent Type A or Type B challenge (Kanistanon et al., 2012). *Francisella* lipid A also has been reported to be unique from other Gram-negative lipid A molecules in that 60–95% can be expressed as “free” lipid A (without core-oligosaccharide or O-antigen) in LVS and *F. novicida* (Wang et al., 2006; Lai et al., 2010; Barker et al., 2014). Whereas, *Yersinia pestis* has been shown to alter the number of acyl chains at different temperatures (Rebeil et al., 2006), *Francisella* modifies its acyl chain length based on temperature changes. At environmental temperatures (18°C), the *F. novicida* LpxD2 enzyme adds 16 carbon-length acyl chains to lipid A, whereas at mammalian temperatures (37°C), the *F. novicida* LpxD1 enzyme adds 18 carbon-length acyl chains. These acyl chain length modifications appear to be important for virulence as a *F. novicida lpxD1* mutant was attenuated in mice via the sub-q route (Li et al., 2012). It has been speculated that these lipid A modifications alter the permeability and fluidity of the outer membrane and may affect outer membrane protein distributions or interactions, which may help *Francisella* adapt to survival in insects and mammalian hosts. Although *lpxD1* and *lpxD2* genes exist in Type A and Type B strains, it is unclear if Type A and Type B strains perform similar carbon chain length modifications based on temperature.

All of the above modifications appear to be important for *Francisella* LPS to minimize recognition by LPS recognition molecules, including LPS-binding protein, MD-2, and TLR4 (Ancuta et al., 1996; Barker et al., 2006; Hajjar et al., 2006). Conventional LPS (i.e., *E. coli*) typically binds to LPS recognition proteins and induces B-cell proliferation. However, only very high doses (~75 μg) of *F. novicida* LPS were able to induce mouse splenocyte proliferation, demonstrating its weak B-cell mitogen activity. Interestingly, similar amounts of LVS LPS did not induce any splenocyte proliferation (Kieffer et al., 2003). As compared to *E. coli* LPS, LVS LPS was not found to be endotoxic by either the *Limulus* amoebocyte assay or in mice sensitivity studies (Sandström et al., 1992). Whereas, monocyes and macrophages produced little to no cytokines in response to LVS LPS, *F. novicida* LPS stimulated macrophages to produce IL-12 and TNFα, highlighting that *F. novicida* is more pro-inflammatory than *F. tularensis* (Sandström et al., 1992; Kieffer et al., 2003).

Despite LPS modifications to avoid various innate immune pathways, LVS LPS was reported to activate the classical complement pathway (Fulop et al., 1993). Complement activation is a double-edged sword for *F. tularensis*, as C3b opsonization is important for host cell phagocytosis but the bacteria must avoid formation of the membrane attack complex. Follow-up studies confirmed that complement component C3 deposits on Schu S4, LVS, and *F. novicida*, but all three strains were complement resistant due to rapid inactivation of complement (conversion to C3bi), which was dependent on the presence of O-antigen (Clay et al., 2008). *F. tularensis* LPS-complement interactions also appear to play a role in macrophage uptake as, in the presence of complement, wild-type LVS was phagocytosed by spacious pseudopod loops, whereas LVS Δ*wbtDEF* or *wbtI*_*G*191*V*_ O-antigen mutants were phagocytosed by tight overlapping pseudopod layers (Clemens et al., 2012). As noted above, since the capsule and O-antigen may share some biosynthetic pathway components, these results are difficult to interpret. However, these results do suggest that O-antigen components, either in the capsule or LPS, may block complement-complement receptor interactions, leading to altered macrophage uptake mechanisms.

Taken together, *Francisella* species have evolved distinct LPS molecules, with a number of structural variations from prototypic LPS, which promote immune evasion. The inert nature of *Francisella* LPS is particularly true for Type A and Type B strains. Further, many of the outlined studies demonstrate that specific LPS modifications contribute to the overall virulence strategy of *F. tularensis*, resulting in high infectivity and severe disease. Despite the low stimulatory capacity of *F. tularensis* LPS, it is interesting to note that this molecule provides species-specific protection. As such, future vaccine development efforts may need to consider incorporating anti-LPS responses that cross-react with both Type A and Type B strains.

## Type IV pili

Type IV pili are important virulence factors for many Gram-negative pathogens, having been shown to play important roles in bacterial adhesion, aggregation, twitching motility, and DNA uptake (Craig et al., 2004; Berry and Pelicic, 2015). *F. tularensis* type IV pili first were reported in 2004 as “horn-like” surface structures that were observed when LVS was grown on agar medium. In contrast, long thin fibers were observed when LVS was grown in liquid medium, indicating that pili-like structures may phenotypically vary depending on culture conditions (Gil et al., 2004). Type IV pili expression also has been reported in Schu S4 and *F. novicida* (Zogaj et al., 2008; Ark and Mann, 2011), suggesting that type IV pili are expressed in other *Francisella* species. However, there also are indications that pilus expression may be strain- or lab-dependent, as two publications have noted that pilus-like structures were not observed on a virulent *F. tularensis* subsp. *holarctica* strain or in *F. novicida* (Forslund et al., 2006; Hager et al., 2006).

Genomic sequencing has confirmed the existence of gene clusters in various *Francisella* species with homology to the type IV pilus genes of *Neisseria* and *Pseudomonas* (Gil et al., 2004; Larsson et al., 2005). The *Francisella* type IV pilus gene clusters appear to contain 14 genes, including various outer membrane, inner membrane, ATPase, and pilin subunit proteins. The surface fiber is composed of between five and six pilin subunits, PilE1–PilE6 (Gil et al., 2004; Ark and Mann, 2011). Whereas all six putative pilin genes are present in Type A strains and *F. novicida*, Type B strains contain various truncations or deletions of *pilE1, pilE2*, or *pilE3* (Ark and Mann, 2011). Indeed, LVS is missing *pilE1* and premature stop codons have been introduced into *pilE2* and *pilE3* (Gil et al., 2004). By comparison, virulent Type B PilE1 (previously described as PilA) was found to form multimers at the bacterial surface and was extensively glycosylated during animal infections, suggesting that type IV pilin glycosylation plays an important role in virulence (Forslund et al., 2006). PilO (also referred to as PglA) was shown to be responsible for PilE1 glycosylation (Egge-Jacobsen et al., 2011). PilE1 has been noted to be required for full virulence of SchuS4 and a virulent Type B strain in sub-q mice infections (Forslund et al., 2006, 2010). The role of PilE1 in *F. tularensis* virulence is further supported by work demonstrating that restoration of *pilE1*, either alone or in combination with FTT0918 (described in Outer Membrane Proteins Section), increases LVS virulence (Salomonsson et al., 2009). Sequence analysis revealed that low-virulence *Francisella* strains contain a long *pilE4* gene (*F. novicida* encodes 316 amino acids; *F. tularensis* LVS encodes 301 amino acids), whereas high-virulence strains contain a truncated *pilE4* gene (subsp. *tularensis* encodes 197 amino acids; subsp. *holarctica* encodes 211 amino acids; Zogaj et al., 2008). Despite these differences in amino acid length, PilE4 appears to be the major pilin component and was required for type IV pilus assembly in Schu S4, LVS, and *F. novicida* (Zogaj et al., 2008; Ark and Mann, 2011). Interestingly, although PilE4 was required for LVS virulence, it was not required for Schu S4 virulence in mice via either sub-q or i.d. infection routes (Ark and Mann, 2011). However, as noted above, the reason for this virulence difference may be due to truncated *pilE4* in Schu S4. The roles of PilE5 and PilE6 in type IV pili assembly and virulence are less clear, as neither were required for pili-like fiber assembly or Schu S4 virulence by either sub-q or i.d. infection routes but both contributed to LVS virulence by both sub-q and i.d. infection routes (Ark and Mann, 2011).

In addition to the surface-exposed pilin subunits, many type IV pili assembly proteins also are required for *Francisella* virulence. Deletion of either *pilC*, an inner membrane protein, or *pilQ*, an outer membrane protein (Huntley et al., 2007), resulted in Schu S4 attenuation in sub-q mice infections (Forslund et al., 2010). Additionally, PilF, the pilus assembly ATPase, and PilT, the pilus retraction ATPase, both were found to be required for LVS virulence in i.d. mice infections (Chakraborty et al., 2008). Interestingly, PilT was not required for Schu S4 virulence in sub-q mice infection studies (Forslund et al., 2010).

In *F. novicida*, individual components of the type IV pili homologous genes have been reported to be involved in a type II-like protein secretion system (Hager et al., 2006; Zogaj et al., 2008). Seven proteins were reported to be secreted by this putative *F. novicida* secretion system, including two chitinases (ChiA and ChiB), a chitin binding protein (CbpA), a protease (PepO), a beta-glucosidase (BglX), and two hypothetical proteins (Fsp58 and Fsp53; Hager et al., 2006). Although a type II-like secretion system have not been reported in virulent *F. tularensis* strains, it is interesting to speculate that the chitinases and CbpA may be important for survival or replication in ticks or other arthropod vectors. In addition, PepO and BglX were found to be regulated by the master virulence regulator MglA, suggesting that they may play roles in damaging host cells (Hager et al., 2006). However, the absence of *pepO* in virulent Type A and Type B *F. tularensis* strains suggests that these findings may be unique to *F. novicida*.

In summary, there are a number of species-specific variations in type IV pili genes, media-dependent variations in type IV pili expression, and conflicting reports about the roles of type IV pili in *F. tularensis* virulence. As such, further studies are needed to better understand *F. tularensis* type IV pili (summarized in Table 2 and Figure 1).

**Table 2:** ***Francisella* genes associated with type IV pili**.

**Gene name**	**Schu S4 locus**	**LVS locus**	***F. novicida* strain U112 locus**	**Function/Location**
*pilC*	FTT1134	FTL0827	FTN1116	Inner membrane
*pilE1 (pilA)*	FTT0890c	absent	FTN0415	Pilin subunit
*pilE2 (pilE)*	FTT0889c	FTL0391	FTN0414	Pilin subunit
*pilE3 (pilV)*	FTT0888c	FTL0390	FTN0413	Pilin subunit
*pilE4*	FTT0861c	FTL0359	FTN0389	Pilin subunit
*pilE5*	FTT0230c	FTL0181	FTN0070	Pilin subunit
*pilE6*	FTT1314c	FTL1475	FTN0664	Pilin subunit
*pilF*	FTT1133	FTL0828	FTN1115	Assembly ATPase
*pilO (pglA)*	FTT0905	FTL0425	FTN1139	PilE1 glycosylase
*pilQ*	FTT1156c	FTL0800	FTN1137	Outer membrane
*pilT*	FTT0088	FTL1771^a^, FTL1770^a^	FTN1622	Retraction ATPase
*chiA*	FTT0715	FTL1521	FTN0627	Secreted protein^b^
*chiB*	FTT1786c	FTL0093	FTN1744	Secreted protein^b^
*cbpA*	FTT1577	FTL0532	FTN1485	Secreted protein^b^
*pepO*	absent	absent	FTN1186	Secreted protein^b^
*bglX*	FTT1069c	FTL0543	FTN1474	Secreted protein^b^
*fsp58*	FTT0580	FTL1331	FTN0753	Secreted protein^b^
*fsp53*	FTT1330	FTL1491	FTN0647	Secreted protein^b^

a *A nonsense mutation in the LVS PilT coding sequence has resulted two loci, FTL1771 and FTL1770*.

b *In F. novicida, individual components of the type IV pili system were reported to be involved in a type II-like secretion system. See text for more details*.

## Secretion systems and secreted proteins

### Type I secretion

Type I secretion systems contribute to bacterial virulence through multi-drug efflux and toxin secretion, typified by the hemolysin secretion system of *E. coli* or TolC secretion system in many Gram-negative bacteria (Piddock, 2006; Thomas et al., 2014; Costa et al., 2015). *F. tularensis* contains two TolC orthologs, TolC and FtlC, both of which are outer membrane-localized (Huntley et al., 2007). Deletion of either *tolC* or *ftlC* from LVS increased sensitivity to antibiotics, dyes, and detergents, suggesting that TolC and FtlC play important roles in *F. tularensis* drug resistance (Gil et al., 2006). Additionally, TolC, but not FtlC, was required for LVS i.d. virulence in mice, indicating that these two TolC orthologs perform non-redundant functions in *F. tularensis* (Gil et al., 2006). A later study found that TolC marginally contributes to Schu S4 i.d. virulence, as *tolC* mutants resulted in 2 day-delayed time-to-death in mice, compared with wild-type infected mice (Kadzhaev et al., 2009). It remains possible that TolC may secrete yet-to-be discovered virulence factors, as LVS *tolC* mutants exhibited *in vivo* dissemination and survival defects, were unable to suppress pro-inflammatory cytokines, and could not inhibit macrophage apoptosis (Platz et al., 2010; Doyle et al., 2014). Another *F. tularensis* outer membrane protein, SilC, originally named due to homology with silver cation efflux proteins in other bacteria, shares sequence homology with *F. tularensis* TolC and FtlC and other bacterial TolC orthologs (Huntley et al., 2007). However, the role of SilC in *F. tularensis* secretion or virulence is unknown.

### Type III and type IV secretion

Type III and Type IV bacterial protein secretion systems are well-known to inject bacterial effectors into host cells, either across the plasma membrane or across internal membranes, promoting bacterial infection and disease (Raymond et al., 2013; Burkinshaw and Strynadka, 2014; Christie et al., 2014). Thus far, functional Type III or Type IV protein secretion systems have not been described in *F. tularensis* and Type III or Type IV secretion system orthologs were not identified in the Schu S4 genome (Larsson et al., 2005). However, subsequent comparative genomic analysis found putative Type III effector and Type IV secretion system homologs in all species of *Francisella*. Interestingly, three of the Type III effector homologs and two of the Type IV secretion system homologs only were present in the avirulent strains (e.g., *F. novicida* and *F. philomiragia*) and were either truncated or absent from Type A, Type B, and *F. tularensis* subsp. *mediasiatica* strains, suggesting that these Type III and Type IV secretion system components may not contribute to human virulence (Champion et al., 2009).

### Type VI secretion

Type VI secretion systems are related to T4-like bacteriophage injection systems and allow translocation of various effector proteins into host or competing bacterial cells (Ho et al., 2014; Russell et al., 2014). Whereas, a putative Type VI protein secretion system reportedly is encoded by the *Francisella* Pathogenicity Island (FPI), *F. novicida* has been almost exclusively used in these descriptions (de Bruin et al., 2007; Barker et al., 2009). The *F. novicida* Type VI secretion system appears to be composed of an outer sheath made up of IglA/IglB heterodimers arranged in a helical cylinder. IglA/IglB sheath assembly was shown to be initiated by various environmental signals, including macrophage uptake, 5% KCl, and altered oxygen tension (Clemens et al., 2015). Structural modeling has suggested that IglC forms the inner tube of the Type VI secretion system and contraction of the sheath has been theorized to drive the inner tube into target membranes (de Bruin et al., 2011; Clemens et al., 2015). VgrG has been suggested to localize to the tip of the Type VI secretion system, forming a trimeric host cell puncturing device (Bröms et al., 2012a). The sheath and tube complex presumably is anchored to the outer membrane by the outer membrane lipoprotein IglE (Robertson et al., 2013; Nguyen et al., 2014). Interestingly, IglE has been shown to interact with the PdpB and DotU inner membrane protein complex and studies suggest that IglE and PdpB form a periplasm-spanning channel (de Bruin et al., 2011; Nguyen et al., 2014). As expected, each of these Type VI structural components have been reported to play important roles in virulence, with IglA and IglB being required for *F. novicida* phagosomal escape, intracellular replication, and virulence in chicken embryo and mouse i.d. infection models (de Bruin et al., 2007; Bröms et al., 2009; Clemens et al., 2015), DotU and VgrG were necessary for LVS or *F. novicida* phagosomal escape and lethal i.d. or i.n. infections in mice (Barker et al., 2009; Bröms et al., 2012a), and IglE was required for *F. novicida* replication in macrophages and lethal i.n. infections in mice (Nguyen et al., 2014).

Despite the above studies noting the existence of a *F. novicida* Type VI secretion system and its role in virulence, the exact secreted effectors are still controversial. In one study, IglC,E,F,I,J, PdpA,E, and VgrG were found to be secreted by LVS into macrophages in a Type VI secretion-dependent manner. However, in that same study, *F. novicida* only was found to secrete IglC,E and PdpA,E indicating that there may be species-specific differences in Type VI secreted effectors (Bröms et al., 2012b). In contrast, a separate study reported that *F. novicida* secreted IglA-J, PdpA,C,E, DotU, and VgrG during macrophage infections (Hare and Hueffer, 2014). Indeed, the role of VgrG in *Francisella* is extremely unclear, with studies indicating that VgrG is a secreted effector (Barker et al., 2009; Clemens et al., 2015), coordinates secretion of other effector molecules (Barker et al., 2009), and/or is a structural component of the Type VI secretion apparatus (de Bruin et al., 2011; Bröms et al., 2012a). Understudied FPI and/or Type VI secretion system components include: IglG and IglI, which were required for LVS phagosomal escape and *in vivo* dissemination (Bröms et al., 2011); and *pdpD* and *anmK*, which were required for *F. novicida* virulence in chicken embryos and mice (Ludu et al., 2008). Interestingly, although *anmK* (predicted chaperone) is found in *F. novicida, anmK* is separated into two smaller ORFs in Type A *F. tularensis* strains, and *anmK* is absent from Type B strains, suggesting that *anmK* is not required for virulence in pathogenic *F. tularensis* strains (Bröms et al., 2010). Additionally, whereas *pdpD* (outer membrane protein in *F. novicida*; Ludu et al., 2008) is present in both Type A *F. tularensis* strains and *F. novicida, pdpD* has major deletions in Type B strains that likely result in two, small non-functional proteins (Bröms et al., 2010).

### Secreted proteins

Various proteins have been reported to be secreted/released by *F. tularensis* but the mechanisms for their secretion are not known. Secretion could occur through one of the secretion systems outlined above, through outer membrane vesicle blebbing, or through yet to be elucidated mechanisms. Some studies have compared the secreted proteome of virulent and attenuated *F. tularensis* strains as a means to identify potential virulence factors. In one study, comparison of culture filtrate proteins from LVS and Schu S4 revealed three proteins detected in higher abundance from Schu S4, including the acid phosphatase AcpA, a beta-lactamase, and hypothetical protein FTT_0484 (Konecna et al., 2010). In another study, culture filtrates from a recent type A clinical isolate and LVS were compared, revealing 12 proteins likely released from both *F. tularensis* strains, including catalase-peroxidase KatG, chaperones DnaK, GroEL, and GroES, superoxide dismutase SodB, and bacterioferritin Bfr. Of these, KatG and DnaK were more prevalent from type A strain culture filtrates, Bfr was detected in multiple isoforms from LVS filtrates, and GroEL was released at equal levels from both *F. tularensis* strains. Interestingly, KatG and GroEL were detected in the cytosol of infected THP-1 cells, indicating that both may be important for intracellular replication (Lee et al., 2006).

AcpA has been found to be either associated with the outer membrane or secreted into culture supernatants by both *F. novicida* and LVS (Mohapatra et al., 2007; Dai et al., 2012). Importantly, AcpA also was isolated from the cytosol of infected macrophages, suggesting a role in virulence (Konecna et al., 2010; Dai et al., 2012). In addition to acid phosphatase activity, multiple functions have been attributed to *F. tularensis* AcpA, including phospholipase activity, lipase activity, ability to inhibit the neutrophil respiratory burst, general roles in intracellular growth, phagosomal escape, and virulence in animals (via i.n. or i.p. infection routes; Reilly et al., 1996; Mohapatra et al., 2007, 2008, 2010; McRae et al., 2010).

KatG has been noted to be important for *in vitro* detoxification of reactive oxygen and nitrogen species in both LVS and Schu S4 (Lindgren et al., 2007). As noted above, since Type A strains were found to express more KatG than LVS (Lee et al., 2006), it is interesting that LVS was found to be more susceptible to reactive oxygen and nitrogen species than virulent Type A or B strains (Lindgren et al., 2007). Despite the hypothetical role of KatG to protect *F. tularensis* from free radical damage, KatG was not found to be necessary for intracellular survival in macrophages for either LVS or Schu S4, but was required for LVS virulence in mice via the i.d. route (Lindgren et al., 2007).

Although far fewer studies have been performed to understand *F. tularensis* SodB, it has been found to confer *in vitro* bacterial resistance to peroxide and the superoxide generating compound paraquat. In addition, SodB was shown to be required for LVS virulence in mice by the i.n. route (Bakshi et al., 2006). The authors of that study proposed multiple modes of activity for SodB, including binding of free iron to limit highly lethal hydroxyl radical production, detoxification of superoxide to limit DNA, protein, and lipid toxicity, and detoxification of superoxide to prevent peroxynitrite formation (Bakshi et al., 2006).

Taken together, TolC and the FPI-encoded type VI-like secretion system appear to be the best candidates for *F. tularensis* secretion systems (Figure 1). Although many proteins have been reported to be released by *F. tularensis* (Table 3), there are major gaps in our understanding of *F. tularensis* secretion mechanisms and secretion machinery. Whereas studies on the *F. novicida* type VI-like secretion system are intriguing and have helped characterize the functions of various *F. novicida* FPI genes (Table 3), parallel studies in virulent *F. tularensis* strains are severely lacking, likely due to duplicate copies of the FPI in Type A and Type B strains. Future studies are needed to better understand *F. tularensis* secretion systems, secreted proteins, the role of the FPI in secretion, and confirm the existence of the Type VI secretion system in virulent *F. tularensis* strains.

**Table 3:** ***Francisella* secretion systems or proteins reported to be secreted**.

**Gene name (if designated)**	**Schu S4 locus**	**LVS locus**	***F. novicida* strain U112 locus**	**Proposed role**
*sodB*	FTT0068	FTL1791	FTN1642	Secreted
*acpA*	FTT0221	FTL0158	FTN0090	Secreted
	FTT0484	FTL1583	FTN0571	Secreted
β-lactamase	FTT0611c	FTL0879	FTN1072	Secreted
*katG*	FTT0721c	FTL1504	FTN0633	Secreted
*ftlC*	FTT1095c	FTL1107	FTN0779	Type I secretion
*silC*	FTT1258	FTL0686	FTN1277	Type I secretion
*dnaK*	FTT1269c	FTL1191	FTN1284	Secreted
*pdpA*	FTT1344, FTT1699	FTL1172, FTL0126	FTN1309	Type VI secretion
*pdpB*	FTT1345, FTT1700	FTL0125, FTL1171	FTN1310	Type VI secretion
*iglE*	FTT1346, FTT1701	FTL0124, FTL1170	FTN1311	Type VI secretion
*vgrG*	FTT1347, FTT1702	FTL0123, FTL1169	FTN1312	Type VI secretion
*iglF*	FTT1348, FTT1703	FTL0122, FTL1168	FTN1313	Type VI secretion
*iglG*	FTT1349, FTT1704	FTL0121, FTL1167	FTN1314	Type VI secretion
*iglH*	FTT1350, FTT1705	FTL0120, FTL1166	FTN1315	Type VI secretion
*dotU*	FTT1351, FTT1706	FTL0119, FTL1165	FTN1316	Type VI secretion
*iglI*	FTT1352, FTT1707	FTL0118, FTL1164	FTN1317	Type VI secretion
*iglJ*	FTT1353, FTT1708	FTL0117, FTL1163	FTN1318	Type VI secretion
*pdpC*	FTT1354, FTT1709	FTL0116, FTL1162	FTN1319	Type VI secretion
*pdpE*	FTT1355, FTT1710	FTL0115, FTL1161	FTN1320	Type VI secretion
*iglD*	FTT1356c, FTT1711c	FTL0114, FTL1160	FTN1321	Type VI secretion
*iglC*	FTT1357c, FTT1712c	FTL0113, FTL1159	FTN1322	Type VI secretion
*iglB*	FTT1358c, FTT1713c	FTL0112, FTL1158	FTN1323	Type VI secretion
*iglA*	FTT1359c, FTT1714c	FTL0111, FTL1157	FTN1324	Type VI secretion
*pdpD*	FTT1360c, FTT1715c	FTL0109, FTL0110, FTL1155, FTL1156	FTN1325	Type VI secretion
*anmK*	FTT1361c, FTT1716c	Absent	FTN1326	Type VI secretion
*bfr*	FTT1441	FTL0617	FTN1410	Secreted
*groES*	FTT1695	FTL1715	FTN1539	Secreted
*groEL*	FTT1696	FTL1714	FTN1538	Secreted
*tolC*	FTT1724c	FTL1865	FTN1703	Type I secretion

## Outer membrane proteins

Because of their surface localization, bacterial outer membrane proteins (OMPs) can play dual roles as virulence factors and as immune targets (Huntley et al., 2008; Confer and Ayalew, 2013; Embers and Narasimhan, 2013; Pore and Chakrabarti, 2013; Cash, 2014; Christodoulides, 2014). For these reasons, many investigators have attempted large-scale proteomic efforts to identify *F. tularensis* OMPs, including the use of biotinylation (Chandler et al., 2015), detergents (Twine et al., 2005b; Dresler et al., 2011), or sodium carbonate (Pavkova et al., 2005, 2006; Janovská et al., 2007) to extract and purify surface proteins. However, in each of those studies, large numbers of cytosolic, periplasmic, or proteins lacking transmembrane domains were identified, questioning the utility of those approaches. Despite these limitations, the following proteins were identified in three or more studies and are bioinformatically-predicted to be OMPs: FTT0077, FTT0087, FTT0188, FTT0209c, FTT0472, FTT0583 (FopA), FTT0721c (KatG), FTT0726c, FTT0842 (Pal), FTT0901 (Tul4-A), FTT1043 (Mip), FTT1103 (DsbA), FTT1441, FTT1483c, FTT1484c, FTT1572c, FTT1591, FTT1676, and FTT1747. To our knowledge, there have been few follow-up studies on these 19 proteins, so little is known about their true subcellular localization or their individual roles in virulence.

Gram-negative bacteria are known to spontaneously release outer membrane vesicles (OMVs) that are highly enriched in OMPs (Bonnington and Kuehn, 2014), thus it is not surprising that OMVs have been collected from *F. novicida* culture supernatants as a means to identify *Francisella* OMPs (Pierson et al., 2011; McCaig et al., 2013). However, mass spectrometry analysis of *F. novicida* OMVs indicated that OMPs compromised only 5–15% of identified proteins. Importantly, spontaneously-released OMVs have not been isolated from virulent *F. tularensis* strains, indicating that OMV shedding may be *F. novicida*-specific. Our laboratory developed and published a rigorous method to generate *F. tularensis* OMVs through spheroplasting and osmotic lysis, followed by OMV isolation using sucrose density gradient centrifugation (Huntley et al., 2007, 2010). This powerful technique resulted in the identification of 17 *bona fide F. tularensis* OMPs (Huntley et al., 2007) and confirmed some of the putative OMPs identified from large-scale proteomic efforts described above (e.g., FopA, KatG, Pal, Tul4-A, Mip, DsbA; Table 4). Additionally, our laboratory has demonstrated the protective potential of *F. tularensis* OMPs to serve as vaccines against Schu S4 challenge (Huntley et al., 2008).

**Table 4:** ***F. tularensis* outer membrane proteins (OMPs)**.

**Gene name (if designated)**	**Schu S4 locus**	**LVS locus**	***F. novicida* strain U112 locus**	**Required for virulence?**
*fslE, srfA*	FTT0025c	FTL1863	FTN1686	Yes
*fsaP*	FTT0119	FTL1658	FTN1596	Yes
*dipA*	FTT0369c	FTL1306	FTN0275	Yes
*fopA*	FTT0583	FTL1328	FTN0756	Yes
*katG*	FTT0721c	FTL1504	FTN0633	
	FTT0825c	FTL0317	FTN0340	
*ompA*	FTT0831c	FTL0325	FTN0346	Yes
*pal*	FTT0842	FTL0336	FTN0357	
*tul4-A*	FTT0901	FTL0421	FTN0427	
	FTT0903	FTL0423	FTN0429	
*tul4-B*	FTT0904	FTL0424	FTN0430	
*yapH-N, fupA, fopC*	FTT0918	FTL0439	FTN0444	Yes
*yapH-C, fupB*	FTT0919	FTL0439	FTN0445	
*mip*	FTT1043	FTL1042	FTN0921	
*tolC-A, ftlC*	FTT1095c	FTL1107	FTN0779	
*dsbA, fipB*	FTT1103	FTL1096	FTN0771	Yes
*pilQ*	FTT1156c	FTL0800	FTN1137	
*SilC*	FTT1258	FTL0686	FTN1277	
*iglE*	FTT1346, FTT1701	FTL0124, FTL1170	FTN1311	Yes
*flpp3*	FTT1416c	FTL0645	FTN1382	Yes
*ftaG*	FTT1573c	FTL0535	FTN1482	
*vacJ*	FTT1591	FTL1637	FTN0322	
*iglE*	FTT1701	FTL1865	FTN1703	
*tolC-B, tolC*	FTT1724c	FTL0105	FTN1734	Yes
	FTT1778c	FTL1863	FTN1686	

Aside from the above noted OMPs, the remainder of this section is devoted to OMPs that have been studied as *F. tularensis* virulence factors. FTT1416c first was identified as a *F. tularensis* membrane protein (Twine et al., 2005b). The LVS homolog, FTL0645, was identified from a transposon library as being required for LVS survival in the mouse lung (Su et al., 2007). Later analysis indicated that FTL0645 (named Flpp3) was a lipoprotein, stimulated TLR2, and reacted with antisera from LVS vaccinees and tularemia patients (Parra et al., 2010). The NMR structure of the Flpp3 Schu S4 homolog, FTT1416c, recently was solved and found to be structurally similar to the Bet v1 family of allergens, suggesting that FTT1416c could be a vaccine candidate (Zook et al., 2015). FTT1416c also was found to contain an internal hydrophobic cavity, suggesting that FTT1416c could be targeted for small molecule inhibitor studies (Zook et al., 2015).

Global transcriptional profiling of Schu S4 from mouse macrophages revealed that FTT0369c (DipA) was upregulated 2- to 3-fold and a Δ*FTT0369c* mutant confirmed that FTT0369c was required for virulence both *in vitro* and *in vivo* (i.n. and i.d. routes tested in mice; Wehrly et al., 2009). More comprehensive studies demonstrated that Schu S4 Δ*dipA* mutants escaped from phagosomes, failed to replicate in the cytosol, and then were targeted to autophagy vacuoles (LAMP1+, LC3+) for clearance (Chong et al., 2012). Biochemical and structural analysis of DipA showed the protein to be outer membrane-localized and surface-exposed in SchuS4. In addition, DipA was found to interact with OMP FopA, suggesting that DipA and FopA form an outer membrane complex (Chong et al., 2013). A more recent study identified DipA as a DsbA substrate and demonstrated that, although DipA contains a putative lipoprotein acylation motif, DipA is not lipidated (Ren et al., 2014).

An OmpA-like protein, FTT0831c in Schu S4 and FTL0325 in LVS, was found to be surface-exposed in LVS and outer membrane-localized in Schu S4 (Mahawar et al., 2012). FTL0325 and FTT0831c are required for macrophage replication and deletion of the gene in each respective strain caused increased proinflammatory cytokine production in macrophages (Mahawar et al., 2012). Ectopic expression of FTT0831c in a mammalian cell line blocked NFκB translocation to the nucleus and suggested that FTL0325/FTT0831c functions to suppress proinflammatory cytokine responses in host cells (Mahawar et al., 2012). The LVS FTL0325 mutant was attenuated in an i.n. mouse infection model, yet still induced high levels of inflammatory cytokines from days one through four post-infection, as compared to wild-type LVS or other LVS mutants (Mahawar et al., 2013). These early pro-inflammatory cytokine responses to FTL0325 were found to correlate with protection against Schu S4 challenge. A later study noted that FTT0831c/FTL0325 mutants had reduced viability in laboratory medium, resulted in small colony phenotypes on agar, were larger and more spherical than wild-type cells, and had an irregular morphology with blebs near the bacterial midpoint and enlarged bacterial tips. Based on these abnormalities, the authors proposed that FTT0831c/FTL0325 acts as a structural protein to link the *F. tularensis* outer membrane to peptidoglycan (Robertson et al., 2014). The authors' results suggested that loss of FTT0831c/FTL0325 resulted in altered cell morphology and decreased viability, likely resulting in increased release or exposure of PAMPs, which may have explained the increased proinflammatory cytokine response previously observed.

FTT0918 first was characterized as a 58-kDa protein required for Schu S4 virulence *in vitro* and *in vivo* (i.d. mice infections; Twine et al., 2005a). Interestingly, in LVS, the FTT0918 homolog is C-terminally truncated and fused in-frame with the N-terminally truncated downstream FTT0919 homolog, resulting in a hybrid protein FTL0439 (Twine et al., 2005a; Huntley et al., 2007). Despite coding differences between Schu S4 and LVS, FTT0918 and FTL0439 both are outer membrane-localized (Huntley et al., 2007). The importance of FTT0918 in *F. tularensis* virulence was further highlighted by a study that reintroduced PilA and FTT0918 into LVS, which restored virulence to wild-type Type B levels (Salomonsson et al., 2009). The *F. novicida* homolog, FTN0444 (named FopC), appears to play similar roles in virulence both *in vitro* and *in vivo* (i.n. mice infections; Nallaparaju et al., 2011). *In vitro* studies demonstrated that, as compared to wild-type *F. novicida, F. novicida* FopC mutants were more susceptible to IFNγ-induced macrophage killing and had decreased membrane stability (Nallaparaju et al., 2011). FTT0918 also has been shown to be important for both siderophore-dependent and siderophore-independent (ferrous) iron acquisition mechanisms in Schu S4 (Lindgren et al., 2009; Ramakrishnan et al., 2012). In this context, FTT0918 has been referred to as FupA, for *F*e *u*tilization protein A (Lindgren et al., 2009). Interestingly, despite its role in iron uptake, *fupA* expression is not regulated by iron limitation (Lindgren et al., 2009; Ramakrishnan et al., 2012). It is well-known that iron is critical for many intracellular pathogens (Cassat and Skaar, 2013; Caza and Kronstad, 2013), thus it is not surprising that FupA-mediated iron uptake was found to be important for Schu S4 virulence both *in vitro* and *in vivo* (sub-q and i.d. routes tested in mice; Lindgren et al., 2009; Ramakrishnan et al., 2012). In LVS, the homologous hybrid protein, FupA/B, has been shown to play similar roles in uptake of both ferrous and siderophore-bound iron (Sen et al., 2010; Ramakrishnan and Sen, 2014). Similarly, FupA/B was found to be important for intracellular growth and for LVS virulence in i.p. infected mice (Sen et al., 2010).

FslE (FTT0025c) is a second *F. tularensis* OMP (Huntley et al., 2007) that has been shown to play a role in iron uptake (Ramakrishnan et al., 2008, 2012; Ramakrishnan and Sen, 2014). In Schu S4, FslE appears to be primarily responsible for siderophore-mediated ferric iron acquisition (Ramakrishnan et al., 2008, 2012). Schu S4 FslE was found to be essential for growth in iron-limiting conditions and, as compared to FupA (FTT0918), FslE expression was increased during growth in iron-limitation (Ramakrishnan et al., 2008, 2012). By comparison, LVS FslE appears to play a secondary role in siderophore-mediatated iron uptake, as FslE was not required for the growth of LVS in iron-limiting conditions (Sen et al., 2010). Interestingly, a Schu S4 *fslE* mutant was not attenuated for intracellular growth or virulence in sub-q-infected mice, but a *fupA–fslE* double mutant was more severely attenuated than a *fupA* mutant in sub-q infected mice, suggesting that FslE and FupA play synergistic roles *in vivo* (Ramakrishnan et al., 2012). Similarly, LVS *fslE* mutants were not attenuated in i.p.-infected mice, but a *fslE-fupA/B* double mutant was more severely attenuated than a *fupA/B* single mutant in i.p.-infected mice, further supporting the synergistic roles of FslE and FupA/B in *F. tularensis* virulence (Sen et al., 2010).

DsbA (FTT1103) was identified as a putative virulence factor through a Schu S4 transposon library screen that identified mutants defective in intracellular growth (Qin and Mann, 2006). A subsequent full-gene deletion confirmed that FTT1103 was required for intracellular survival, phagosomal escape, and virulence in mice (i.n. and sub-q routes tested; Qin et al., 2009). A number of follow-up studies have implicated DsbA (FTT1103 in Schu S4, FTL1096 in LVS, also referred to as FipB) as an essential *F. tularensis* virulence factor (Qin and Mann, 2006; Thakran et al., 2008; Qin et al., 2009, 2011, 2014; Straskova et al., 2009; Schmidt et al., 2013; Senitkova et al., 2015). However, given the well-known function of bacterial DsbA proteins to form disulfide bonds in various substrates, including true virulence factors, it seems unlikely that *F. tularensis* DsbA directly promotes host cell binding, invasion, intracellular survival, or other virulence functions. Further, *F. tularensis* DsbA is unusual in that it is outer membrane-localized and lipidated, as compared to the periplasmically-localized DsbA in other bacteria (Huntley et al., 2007; Thakran et al., 2008). *F. tularensis* DsbA lipidation has been demonstrated to stimulate the TLR2/TLR1 heterodimer and induce proinflammatory chemokine expression (Thakran et al., 2008). Additionally, *F. tularensis* DsbA has been reported to be glycosylated, although the role of this glycosylation is unknown (Balonova et al., 2012). Finally, *F. tularensis* DsbA is distinct from other bacterial DsbA orthologs in that it contains an N-terminal Forskolin-binding protein-N (FKBP-N) dimerization domain as well as a C-terminal DsbA-like domain (Qin et al., 2011). The function of the DsbA N-terminal FKBP-N domain is somewhat controversial, as this domain has been reported to possess isomerase and chaperone activities, was reported to be required for DsbA dimerization, and was shown to be required for virulence in mice by either i.n. or sub-q infection routes (Qin et al., 2014; Senitkova et al., 2015). Conversely, other studies have reported that the FKBP-N domain does not possess chaperone activity, is not required for dimerization, and is not required for virulence in mice by either i.n. or sub-q infection routes (Schmidt et al., 2013; Qin et al., 2014). More thorough analyses are needed to clarify the true function of the DsbA FKBP-N domain. The C-terminal DsbA-like domain has been more carefully studied, consistently demonstrating that the thioredoxin-like CXXC active site motif is required for *in vitro* and mouse virulence (i.n. and sub-q routes tested; Qin et al., 2011; Schmidt et al., 2013). The DsbA-like domain also has been shown to possess disulfide oxidoreductase activity (Straskova et al., 2009; Schmidt et al., 2013; Qin et al., 2014). Work by our laboratory recently revealed that *F. tularensis* DsbA is a bi-functional protein, possessing both oxidoreductase and isomerase activities to correctly form complex disulfide bonds in over 50 substrates, including some of the above noted virulence factors (e.g., DipA, FopA, and FTT0831c), type IV pilus components, pathogenicity island proteins, previously described OMPs, and 25 hypothetical proteins (many of which are predicted OMPs). As proof-of-principal that these newly-identified DsbA substrates were true virulence factors, we demonstrated that at least two novel proteins, FTL1548 and FTL1709, were required for LVS i.n. infection of mice (Ren et al., 2014).

Among other *F. tularensis* OMPs that have been studied, FsaP (FTL1658) was shown to be a surface-exposed OMP important for LVS adherence to epithelial cells (Melillo et al., 2006). Overexpression of FsaP in *E. coli* increased bacterial adherence to epithelial cells, confirming the LVS findings. Interestingly, despite 98% sequence identity to its LVS and Schu S4 (FTT0119) homologs, the *F. novicida* FsaP (FTN1596) homolog has an amino acid substitution in its signal sequence that appears to affect FsaP localization and function, as *F. novicida* FsaP was not outer membrane-localized and *F. novicida* did not adhere to epithelial cells as well as LVS (Melillo et al., 2006). Already highlighted in the Secretion Systems and Secreted Proteins Section above are two FPI members and reported components of a Type VI-like secretion system, outer membrane lipoprotein IglE (FTT1346/FTT1701) and putative outer membrane protein PdpD (outer membrane localization only shown in *F. novicida;* Ludu et al., 2008). Also highlighted in the Secretion Systems and Secreted Proteins Section were OMPs and TolC homologs TolC (FTT1724c), FtlC (FTT1095c), and SilC (FTT1258). Of these, only TolC has been shown to be involved in virulence.

In summary, the large number of reported *F. tularensis* OMPs and studies to understand their function (Table 4) highlight the importance of OMPs as potential virulence factors and vaccine targets (Figure 1). Future OMP identification and characterization studies will help to better understand the extreme virulence of *F. tularensis*.

## Periplasmic and inner membrane proteins

In Gram-negative bacteria, the periplasm and inner membrane (IM) compartmentalize and protect the cytoplasmic contents from the harsh external environment. In *E. coli*, more than 300 periplasmic proteins perform a variety of functions, including folding proteins into their native confirmation, sensing unfolded or damaged proteins (chaperones), degrading proteins, or binding/transporting sugars, amino acids, and other nutrients between the OM and IM (Merdanovic et al., 2011; Goemans et al., 2014). The IM is vitally important for many processes, including energy production, lipid biosynthesis, transport of sugars, amino acids, and other nutrients into the cytoplasm, secretion of proteins out of the cytoplasm, and signal transduction (Silhavy et al., 2010; Luirink et al., 2012). Despite the essential nature of these proteins for bacterial viability, only a few *F. tularensis* periplasmic or IM proteins have been studied and correlated with virulence.

SodC (FTL0380) is predicted to be a periplasmic protein related to the copper/zinc-containing superoxide dismutase family. LVS SodC was found to be important for peroxide and superoxide resistance *in vitro*, but was not involved in reactive nitrogen resistance. In IFN-γ-activated macrophages, *sodC* mutants were attenuated for macrophage growth and were found to be partially attenuated in a mouse i.n. infection model (Melillo et al., 2009).

RipA (FTL1914), an IM protein, first was identified by screening a LVS transposon library for mutants that failed to replicate in alveolar epithelial cells (Fuller et al., 2008). Although RipA is conserved among all sequenced *Francisella* strains, it has few orthologs in other bacteria, and its function is not completely understood. *In vitro* analyses demonstrated that the Δ*ripA* mutant invaded macrophages and epithelial cells as efficiently as wild-type LVS, but the Δ*ripA* mutant had significant survival defects in both cell types. More specifically, the Δ*ripA* mutant escaped from phagosomes and re-entered autophagy-like vacuoles as efficiently as wild-type LVS, but was unable to survive in the host cell cytoplasm. *In vivo*, the Δ*ripA* mutant failed to replicate in mouse lungs following i.n. challenge and was severely attenuated for dissemination to livers and spleens of infected mice (Fuller et al., 2008). pH appears to play a role in the Δ*ripA* cytosolic replication defect, as the Δ*ripA* mutant replicated similar to wild-type LVS in media at pH 6.2, but the Δ*ripA* mutant replicated poorly at pH 7.5 (similar to the pH in the macrophage cytosol). Further, *ripA* transcription was found to be upregulated at pH 7.5, compared with gene expression at pH 5.5 (Fuller et al., 2009). Two studies noted that RipA plays a role in immune evasion, as the Δ*ripA* mutant induced inflammasome activation, including IL-1β, IL-18, and TNF-α, and cell death in macrophages (Huang et al., 2010; Mortensen et al., 2012). However, in *F. novicida*, a Δ*ripA* mutant was found to possess aberrant cell morphology and lysed more readily inside macrophages than wild-type *F. novicida*, suggesting that Δ*ripA* mutants induced inflammasome activation due to PAMP release (Peng et al., 2011). A separate study showed that RipA interacts with and modulates LpxA, a component of the Lipid A biosynthesis pathway (Miller et al., 2014). Taken together, it appears that RipA may not actively suppress immune responses, but instead may modulate lipid A/LPS expression inside host cells and promote intracellular survival (Miller et al., 2014).

The KdpDE two component signaling system has been shown to be important for potassium ion homeostasis and virulence in diverse bacterial species (Freeman et al., 2013). All *F. tularensis* strains encode the IM-bound sensor kinase KdpD (FTT1736c, FTL1879, FTN1715). However, the tandemly-arranged response regulator KdpE (FTN1714) only is present in *F. novicida* and not in virulent *Francisella* strains. *F. novicida* KdpD was shown to be the primary kinase that phosphorylated the orphan response regulator PmrA (FTT1557c, FTL0552, FTN1465) and this phosphorylation increased PmrA binding to *pmrA* and *pdpD* promoters. Phosphorylation of PmrA also was found to be important for virulence in macrophages and i.n. infection of mice (Bell et al., 2010). Other studies have confirmed the role of *F. novicida* KpdD for *in vivo* virulence, including in *Drosophila* (Moule et al., 2010) and mice (sub-q and i.p. routes tested; Weiss et al., 2007) infection models. *F. novicida* Δ*kdpD* mutants also were found to be more sensitive to reactive oxygen species, but not antimicrobial peptides *in vitro* (Moule et al., 2010).

QseBC is a two-component signaling system found in many bacterial pathogens that responds to host stress hormones epinephrine and norepinephrine, as well as the bacterial signal AI-3 (Clarke et al., 2006; Curtis et al., 2014; Weigel and Demuth, 2015). In *F. tularensis*, the IM sensor kinase QseC (FTT0094c, FTL1762, FTN1617) is not transcriptionally linked to a response regulator, although homology searches suggest that PmrA (FTT1557c, FTL0552, FTN1465; described above) may be a QseB homolog (Durham-Colleran et al., 2010). *F. novicida* QseC mutants were found to be attenuated in *Drosophila* (Moule et al., 2010) and mice (i.p. and sub-q routes tested; Weiss et al., 2007) infection models. In LVS, a Δ*qseC* mutant was extremely serum sensitive, could no longer synthesize high molecular mass O-polysaccharide in its LPS, and was attenuated for macrophage growth and virulence in mice via the sub-q route (Mokrievich et al., 2010). QseC also has been reported to be important for *F. novicida* biofilm production (Durham-Colleran et al., 2010). In a separate study, maprotiline, an FDA-approved norepinephrine uptake inhibitor, was found to inhibit *F. novicida* biofilm production in a QseC dependent manner and prolonged survival in a mouse i.n. infection model (Dean and van Hoek, 2015). Due to the broad distribution of QseC across many pathogenic bacteria, QseC is an attractive target for anti-virulence drug development efforts (Rasko et al., 2008; Curtis et al., 2014; Dean and van Hoek, 2015). Indeed, Schu S4 QseC was found to be upregulated during mammalian infection and a small-molecule inhibitor of QseC, LED209, decreased expression of many *F. tularensis* virulence genes and attenuated intracellular survival and virulence in mice in an i.n. infection model (Rasko et al., 2008; Curtis et al., 2014).

Four other IM proteins, LpxE (Wang et al., 2004), LpxF (Wang et al., 2006, 2007), KdoH1, and KdoH2 (Zhao and Raetz, 2010), have been implicated in *F. tularensis* virulence and antimicrobial resistance, but their roles in LPS biosynthesis and modification suggest an indirect role. Finally, although SecY, LolC, and Cht1 were shown to be IM-localized in Schu S4 and LVS (Huntley et al., 2007, 2010) their roles in virulence are not known.

In summary, given the fairly limited number of studies performed to identify *F. tularensis* periplasmic and IM proteins and examine their roles in virulence (Table 5), more emphasis should be devoted to studying these potentially-important proteins. As noted in the beginning of this section, given the large number of periplasmic proteins in other Gram-negative bacteria and critical role of IM proteins in bacterial survival, periplasmic and IM proteins may be an understudied area with great potential to reveal new *F. tularensis* virulence mechanisms.

**Table 5:** ***F. tularensis* periplasmic and inner membrane proteins (IMPs)**.

**Gene name (if designated)**	**Schu S4 locus**	**LVS locus**	***F. novicida* strain U112 locus**	**Bacterial location**
*qseC*	FTT0094c	FTL1762	FTN1617	Inner membrane
*ripA*	FTT0181c	FTL1914	FTN0157	Inner membrane
*secY*	FTT0345	FTL0256	FTN0259	Inner membrane
*kdoH2*	FTT0398c	FTL0464	FTN0494	Inner membrane
*kdoH1*	FTT0399c	FTL0465	FTN0495	Inner membrane
*lolC*	FTT0404	FTL0474	FTN0502	Inner membrane
*cht1*	FTT0715	FTL1521	FTN0627	Inner membrane
*sodC*	FTT0879	FTL0380	FTN0405	Periplasm
*lpxE*	FTT0891	FTL0393	FTN0416	Inner membrane
*lpxF*	FTT1643c	FTL1815	FTN0286	Inner membrane
*kdpD*	FTT1736c	FTL1879	FTN1715	Inner membrane

## Summary and perspectives

Since 2001, there has been a surge in research studies conducted on *F. tularensis* and its non-pathogenic relative *F. novicida*. Whereas, these studies have provided a wealth of information about many previously unstudied *F. tularensis* hypothetical proteins and have revealed novel virulence strategies, major questions still remain about the true role of many of these putative virulence factors in Type A and Type B virulent strains. In this review, we focused on components of the *F. tularensis* envelope that have been reported to contribute to immune evasion, intracellular replication and survival, and mammalian virulence. Since the envelope is the outermost layer of *F. tularensis*, many studies have aimed to identify and characterize envelope components, given their potential surface exposure and likely direct interaction with host cell molecules. First, the *F. tularensis* capsule has been shown to be identical to the O-antigen, blocks both IgM and complement component C3 binding, and is required for *in vitro* and *in vivo* virulence of many *F. tularensis* strains, including Schu S4 (Figure 1). However, capsule expression appears to be variable and overlap between capsule and LPS synthesis pathways (Table 1) brings into question if *F. tularensis* capsule is real or if the bacterium simply expresses free O-antigen that aggregates near the surface. These questions highlight the need for future studies to better understand capsule. Second, *F. tularensis* has evolved a unique LPS with many structural variations from typical Gram-negative LPS, including fewer acyl chains (tetraacylated), longer fatty acid chains (16–20 carbons), and reduced or no lipid A phosphorylation (Figure 1). Importantly, *F. tularensis* also is able to subtly alter expression or components of its LPS, including O-antigen and lipid A, which affect outer membrane stability and immunogenicity. Together, these LPS alterations affect animal virulence, immune activation/evasion, and the enigmatic blue-gray colony phenotype that plagues the field. As noted above, since *F. tularensis* capsule and LPS appear to share some biosynthetic pathways (Table 1) and serum resistance has been linked to both capsule and LPS, future studies are needed to resolve how these envelope components, either separately or cooperatively, contribute to serum resistance. Third, type IV pili are responsible for thin fibers or horn-like protrusions observed on the surface of many *F. tularensis* strains and have been shown to be required for *F. tularensis* virulence (Figure 1). Although there has been some speculation that components of *F. novicida* type IV pili may be involved in a putative type II-like secretion system, these preliminary findings have not been confirmed in virulent *F. tularensis* strains. Interestingly, despite the well-known role of type IV pili in adhesion, aggregation, twitching motility, and DNA uptake in other bacteria, similar functions have not been attributed to *F. tularensis* type IV pili. Whereas, a number of studies have demonstrated that *F. tularensis* type IV pili are important for Type A and Type B virulence, more detailed studies are needed to elucidate the mechanisms by which type IV pili interact with or damage the host. Fourth, a number of investigators have examined culture filtrate proteins from various *F. tularensis* strains and identified proteins that presumably are secreted (Table 3). Indeed, whereas there is much interest in identifying *F. tularensis* secretion systems and secreted proteins to help explain how the bacterium blocks phagosome-lysosome fusion or escapes from the phagosome, there still is a dearth of information about *F. tularensis* secretion. One possibility is that the *F. tularensis* TolC orthologs, including TolC, FtlC, and SilC (Table 3), function as a Type I secretion system to release effector molecules (Figure 1). Of these, TolC has been shown to play a role in Schu S4 and LVS virulence. Another possibility is that the *F. novicida* FPI-encoded Type VI-like secretion system delivers effector proteins to host cells (Figure 1). However, the existence of a Type VI-like secretion system in virulent *F. tularensis* has not been reported. Fifth, at least 10 OMPs have been shown to be required for *F. tularensis* virulence (Table 4 and Figure 1). Although OMP functions are diverse, these studies highlight the importance of identifying and characterizing OMP function to better understand *F. tularensis* virulence. Sixth, although few studies exist on *F. tularensis* periplasmic or inner membrane proteins, the existing studies indicate that the periplasm plays a key role in detoxifying free radicals and the inner membrane contains two component signaling systems important for altering gene expression during infection (Figure 1).

In conclusion, the *F. tularensis* envelope is a dynamic and robust outer structure that protects the bacterium from immune recognition and clearance, facilitates host cell invasion and survival, and promotes infection and disease. Despite many advances over the decades and in recent years, much more work is needed to better understand the broad host range, environmental persistence, and extreme virulence of this complex pathogen.

## Author contributions

HR and JH reviewed all listed references, compiled information for all tables and figures, and wrote the manuscript.

### Conflict of interest statement

The authors declare that the research was conducted in the absence of any commercial or financial relationships that could be construed as a potential conflict of interest.
